# The pharmacology and therapeutic role of cannabidiol in diabetes

**DOI:** 10.1002/EXP.20230047

**Published:** 2023-07-12

**Authors:** Jin Zhang, Cong Lin, Sha Jin, Hongshuang Wang, Yibo Wang, Xiubo Du, Mark R. Hutchinson, Huiying Zhao, Le Fang, Xiaohui Wang

**Affiliations:** ^1^ Department of Geriatrics The First Hospital of Jilin University Changchun People's Republic of China; ^2^ State Key Laboratory of Natural and Biomimetic Drugs Peking University Beijing People's Republic of China; ^3^ Laboratory of Chemical Biology, Changchun Institute of Applied Chemistry Chinese Academy of Sciences Changchun People's Republic of China; ^4^ School of Applied Chemistry and Engineering University of Science and Technology of China Hefei People's Republic of China; ^5^ Shenzhen Key Laboratory of Marine Biotechnology and Ecology College of Life Sciences and Oceanography Shenzhen University Shenzhen People's Republic of China; ^6^ Discipline of Physiology Adelaide Medical School University of Adelaide The Commonwealth of Australia Adelaide Australia; ^7^ ARC Centre for Nanoscale BioPhotonics University of Adelaide The Commonwealth of Australia Adelaide Australia; ^8^ Department of Neurology The China‐Japan Union Hospital of Jilin University Changchun People's Republic of China; ^9^ Beijing National Laboratory for Molecular Sciences Beijing People's Republic of China

**Keywords:** activity‐based protein profiling, cannabidiol, diabetes, drug discovery, thermal proteome profiling

## Abstract

In recent years, cannabidiol (CBD), a non‐psychotropic cannabinoid, has garnered substantial interest in drug development due to its broad pharmacological activity and multi‐target effects. Diabetes is a chronic metabolic disease that can damage multiple organs in the body, leading to the development of complications such as abnormal kidney function, vision loss, neuropathy, and cardiovascular disease. CBD has demonstrated significant therapeutic potential in treating diabetes mellitus and its complications owing to its various pharmacological effects. This work summarizes the role of CBD in diabetes and its impact on complications such as cardiovascular dysfunction, nephropathy, retinopathy, and neuropathy. Strategies for discovering molecular targets for CBD in the treatment of diabetes and its complications are also proposed. Moreover, ways to optimize the structure of CBD based on known targets to generate new CBD analogues are explored.

## Graphical abstract

Schematic illustration of the therapeutic role and drug development of cannabidiol (CBD) for diabetes. This article comprehensively summarizes the pharmacology and therapeutic role of CBD for diabetes and its complications. Furthermore, the strategies for identifying molecular targets of CBD and developing CBD analogues with greater therapeutic potency have been proposed.

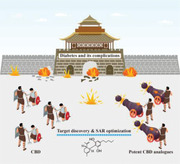

## 1 INTRODUCTION

Cannabis has high economic and medicinal value, as it is widely used in functional health food, cosmetics, medicine, and other fields.^[^ ^1^, ^2^ ^]^ In recent years, the medical uses of cannabinoids, have been gaining great interest in treating a range of medical conditions.^[^ ^1^, ^2^ ^]^ Cannabidiol (CBD) is one of the main pharmacologically active phytocannabinoids in cannabis and has received widespread attention because of its multiple pharmacological effects and its lack of psycho‐activity.^[^ ^3^ ^]^

**FIGURE 1:**
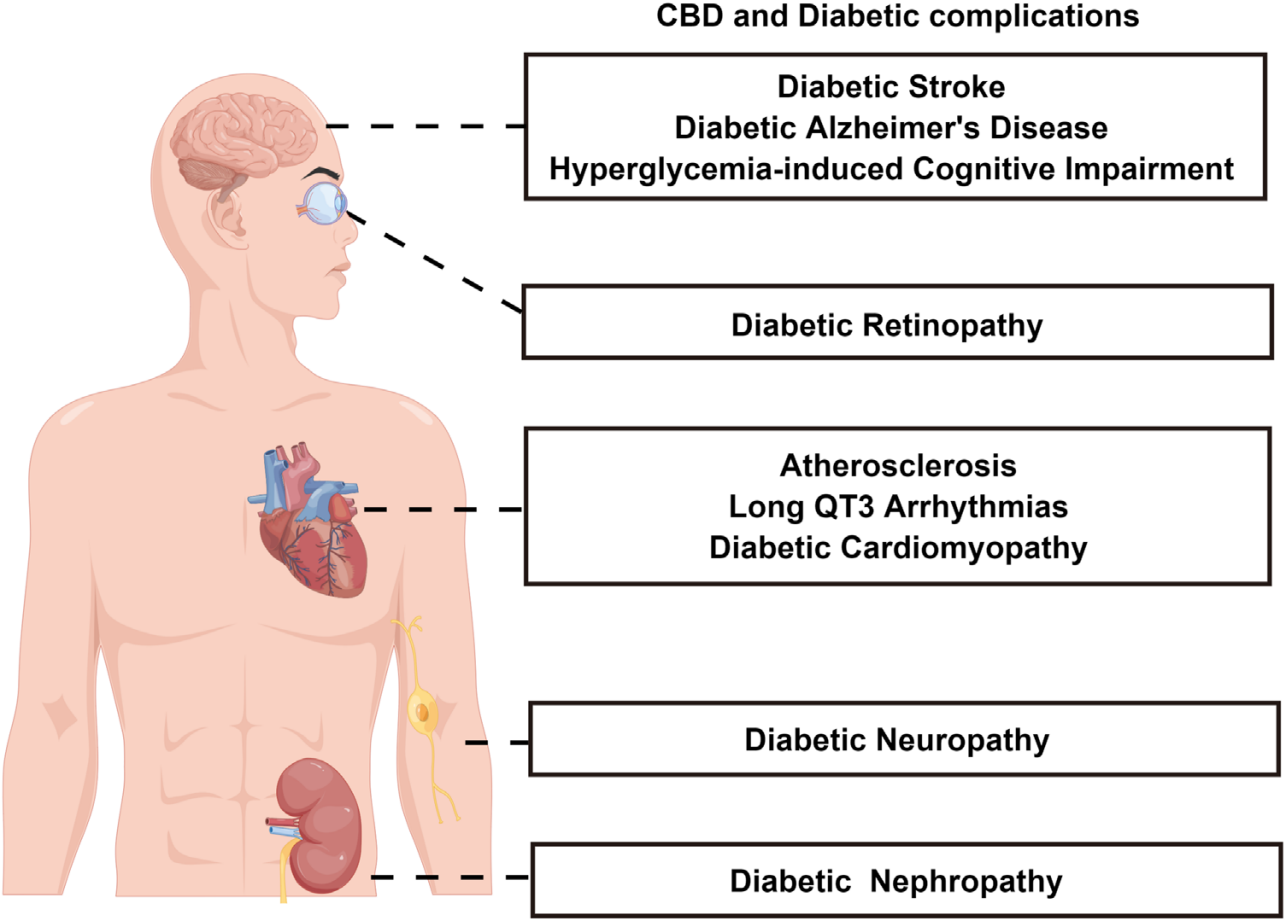
Schematic illustration of the diabetic complications that can be treated by CBD (By Figdraw).

As the seventh most fatal disease worldwide,^[^ ^4^ ^]^ diabetes is a metabolic disorder that affects 463 million adults and is expected to rise to 592 million by 2035 and to 700 million by 2045.^[^ ^5^ ^]^ Diabetes can damage many organs of the body, leading to abnormal kidney function, loss of vision, neuropathy, cardiovascular and cerebrovascular diseases, and other symptoms (**Figure** **1**).^[^ ^6^ ^]^ Diabetes is characterized by hyperglycemia, which is caused by the lack of insulin or insulin resistance due to the autoimmune destruction of pancreatic islet cells. Depending on its pathogenesis, it is currently clinically classified into type 1 diabetes (T1D) and type 2 diabetes (T2D).^[^ ^7^ ^]^

T1D (insulin‐dependent) is characterized by a deficiency of insulin secretion, and usually, a variety of autoimmune antibodies can be detected in patients’ blood. These abnormal autoantibodies can damage pancreatic β‐cells through immune‐mediated damage, preventing them from secreting insulin properly.^[^ ^7^, ^8^ ^]^ T2D (non‐insulin‐dependent) is characterized mainly by a progressive lack of insulin secretion from pancreatic cells and resistance to insulin action by surrounding target tissues,^[^ ^7^, ^9^ ^]^ which accounts for 2%–4% of people and is more common in men.

Studies have shown that CBD can decrease the incidence of diabetes, pancreatic inflammation, and β‐cell destruction in a T1D mouse model.^[^ ^10^, ^11^ ^]^ CBD can shift the T1D‐associated immune response from type 1 to type 2 lymphocyte dominance, which leads to decreased levels of pro‐inflammatory cytokines, such as interferon γ (IFN‐γ) and tumor necrosis factor α (TNF‐α).^[^ ^11^ ^]^ CBD also improves early diabetic symptoms in a mouse model of T1D.^[^ ^12^ ^]^ It has been found that CBD could reduce weight gain and increase energy expenditure by upregulating the expression of protein kinase B, mitochondrial uncoupling protein 2, and glucose transporter protein 2 in pancreatic β‐cells in a rat model of diet‐induced obesity.^[^ ^13^ ^]^ CBD‐treated adipocytes and preadipocytes promote higher levels of glucose uptake, reduce fat accumulation and are more effective in the reversal of insulin resistance.^[^ ^14^ ^]^ Therefore, CBD has therapeutic potential for diabetes and its complications. In this review, we comprehensively discuss the pharmacological roles of CBD in diabetes and its effects on various complications such as diabetic cardiovascular dysfunction, nephropathy, retinopathy, and neuropathy. Moreover, the discovery of molecular target(s) for CBD in the treatment of diabetes and its complications as well as further CBD modification will be also highlighted.

## 2 CBD AND DIABETIC COMPLICATIONS

Diabetes has upwards of 100 complications. The main pathological change in diabetic complications is damage to the vascular wall. Atherosclerosis is the most common macrovascular complication associated with diabetes. It is a condition where plaque builds up inside the arteries, which can lead to blockages and reduce blood flow to vital organs such as the heart and brain. This can increase the risk of heart attacks, strokes, and peripheral arterial disease. Diabetes can also cause microvascular complications, which affect the small blood vessels in the body, leading to lesions in the kidneys, retina, and peripheral nerves.^[^ ^15^ ^]^ The hyperglycemia‐induced increased reactive oxidative products (ROS) of mitochondrial production^[^ ^16^, ^17^ ^]^ is a common underpinning of chronic complications of diabetes. In addition, inflammatory processes caused by monocyte/macrophage infiltration also play a role in target organ damage.^[^ ^18^ ^]^ The direct intervention of CBD in the pathogenesis of diabetic complications makes it an active therapeutic agent for chronic complications of diabetes as well as target organ damage. The possible mechanisms of CBD for the treatment of diabetic complications are shown in **Table** **1**, which will be thoroughly discussed in the following sub‐sections.

**TABLE 1:** Mechanism of CBD for diabetic complications.

Diabetes complications	The therapeutic mechanism of CBD	Ref.
Cardiovascular complications	Inhibition of inflammation Inhibition of oxidative stress Inhibition of protein phosphorylation Activation of adenosine A1 receptors Increased expression of RISK PI3K/AKT and MAPK/ERK pathways	[20, 22, 25, 27, 29, 30]
Diabetic nephropathy	Contradictions exist. To be further verified	[32, 33, 34, 35]
Diabetic retinopathy	Blocking the activation of NMDARs Inhibition of p38‐MAPK phosphorylation	[37, 38]
Diabetic neuropathy	Activation of 5‐HT_1A_ receptors Inhibition of spinal microglia activation Inhibition of p38‐MAPK phosphorylation	[41, 42]
Diabetic brain injury	Activation of TRPV1 Activation of PPAR‐γ Inhibition of inflammation Inhibition of oxidative Stress Inhibition of acetylcholinesterase	[57, 58, 63, 64]

Abbreviations: 5‐HT_1A_: 5‐hydroxytryptamine 1A receptors; AKT: protein kinase B; ERK: extracellular regulated protein kinases; MAPK: mitogen‐activated protein kinase; NMDARs: *N*‐methyl‐d‐aspartate receptors; PI3K: phosphatidylinositol 3 kinase; PPAR‐γ: peroxisome proliferator‐activated receptor γ; RISK: reperfusion injury salvage kinase; TRPV1: transient receptor potential vanilloid type 1.

### 2.1 Cardiovascular complications

Cardiovascular complications of diabetes, including atherosclerosis, diabetic cardiomyopathy, and arrhythmias, are the major causes of mortality and morbidity in the diabetic population.^[^ ^19^ ^]^ Coronary atherosclerotic heart disease (CHD) caused by diabetes is more serious, often with larger myocardial infarcts, more infarcts through the wall, poorer prognosis, and a higher death rate, when compared to CHD caused by other factors. Studies have confirmed that CBD reduces inflammation and oxidative stress in vascular endothelial cells, downregulates the expression of adhesion factors, and inhibits transendothelial migration and adhesion of monocytes to endothelial cells.^[^ ^20^ ^]^ This prevents the disruption of endothelial barrier function and inhibits atherosclerosis formation,^[^ ^20^ ^]^ which is independent of cannabinoid receptor type 1 (CB1) and cannabinoid receptor type 2 (CB2) receptors. Moreover, CBD can exert a cardioprotective effect by relieving the infarcted myocardium in acute myocardial ischemia,^[^ ^21^, ^22^, ^23^, ^24^ ^]^ and ischemia reperfusion‐induced ventricular arrhythmias through the activating of adenosine A1 receptors and the modulation of reperfusion injury salvage kinase (RISK)/phosphatidylinositol 3 kinases (PI3K)/protein kinase B (AKT) and mitogen‐activated protein kinase (MAPK)/extracellular regulated protein kinases (ERK) pathways.^[^ ^22^, ^25^ ^]^

Diabetic cardiomyopathy is a unique prodrome that can lead to heart failure in diabetic patients, which is independent of the macrovascular complications of diabetes.^[^ ^26^ ^]^ CBD attenuates myocardial fibrosis and improves myocardial dysfunction by inhibiting diabetes‐induced activation of nuclear factor kappa‐B (NF‐κB) and MAPK, oxidative‐nitrative stress, inflammation, and cell death in animal models of diabetic cardiomyopathy.^[^ ^27^ ^]^ There is a strong correlation between diabetes and long QT syndrome considering that hyperglycemia can influence the function of myocardial voltage‐gated sodium channel 1.5 (Nav1.5).^[^ ^28^ ^]^ CBD can block Nav1.5 by inhibiting protein phosphorylation of protein kinases A and C, thereby ameliorating the resulting long QT3 arrhythmias and subsequent clinical conditions such as ventricular fibrillation and sudden death.^[^ ^29^, ^30^ ^]^

### 2.2 Diabetic nephropathy

Diabetic nephropathy is a major cause of end‐stage renal failure. Oxidative stress, inflammation, and fibrosis play key roles in the development of diabetic nephropathy, which leads to chronic kidney disease characterized by thylakoid expansion, glomerular basement membrane thickening, and glomerulosclerosis.^[^ ^31^ ^]^ The clinical features are the increased glomerular permeability to proteins and the sustained decline in renal function.^[^ ^31^ ^]^ CBD is able to attenuate the infiltration of inflammatory cells in the kidney, but does not prevent kidney fibrosis and even further aggravates kidney damage and renal dysfunction in type 1 diabetic mice.^[^ ^32^ ^]^ This conclusion contradicts the results of two clinical trials. One phase I clinical trial demonstrated that a single oral dose of CBD (200 mg) had no effect on renal function and was well tolerated by subjects with varying degrees of renal insufficiency.^[^ ^33^ ^]^ The other clinical trial demonstrated that long‐term administration of CBD (100 mg) had no corresponding renal side effects.^[^ ^34^ ^]^ For therapeutic purposes, CBD has been reported to reduce nephrotoxicity caused by the chemotherapeutic drug cisplatin, improve renal function and reduce tubular necrosis and apoptosis.^[^ ^35^ ^]^ Therefore, further investigations are warranted for clarifying the role of CBD in diabetic nephropathy.

### 2.3 Diabetic retinopathy

Diabetic retinopathy is one of the serious microvascular complications caused by diabetes. It occurs when high levels of blood sugar damage the small blood vessels in the retina. This damage can cause the blood vessels to leak, swell, or even close off completely, which can lead to vision loss and blindness if left untreated.^[^ ^34^ ^]^ Early diabetic retinopathy is characterized by the development of microvascular abnormalities in the retina, including microaneurysms and microangiopathy. These can lead to leakage of blood and fluid into the surrounding tissue, causing macular edema and intraretinal hemorrhage. If left untreated, these changes can progress to more advanced stages of diabetic retinopathy, including proliferative diabetic retinopathy. In proliferative diabetic retinopathy, new blood vessels grow on the surface of the retina, which are fragile and prone to bleeding that can cause vitreous or pre‐retinal hemorrhage.^[^ ^36^ ^]^ The therapeutic effect of CBD on experimental diabetic retinopathy has been demonstrated. The administration of CBD enables a significant reduction in neurotoxicity and inflammatory response by inhibiting p38 MAPK activity, therefore protecting the blood‐retinal barrier.^[^ ^37^ ^]^ In addition, CBD may attenuate glutamine excitotoxicity via *N*‐methyl‐d‐aspartate receptors (NMDARs) in retinal neurons by reducing diabetes‐induced tyrosine nitration.^[^ ^38^ ^]^

### 2.4 Diabetic neuropathy

Diabetic neuropathy is one of the most common chronic complications of diabetes, with approximately half of all diabetic patients suffering from this complication.^[^ ^39^ ^]^ The associated abnormalities in glucose metabolism and axonal degeneration secondary to endoneurial microvascular injury are involved in both central and peripheral nerves.^[^ ^40^ ^]^ Clinical manifestations of diabetic neuropathy include painful neurological symptoms such as spontaneous tingling, stabbing or burning sensations, nociceptive hyperalgesia and ectopic pain, loss of sensation, and eventually foot ulceration and amputation.^[^ ^39^, ^40^ ^]^

The treatment of diabetic neuropathy remains a major clinical challenge. The current treatment is still the control of blood glucose levels and the application of analgesic drugs, which commonly include antidepressants and anticonvulsants, but these are less effective.^[^ ^39^, ^40^ ^]^ Spinal microglial activation and increased p38‐MAPK phosphorylation play important roles in the development of neuropathic pain in diabetes. CBD has been found to prevent microglial accumulation and activation in the dorsal spinal cord and inhibit the phosphorylation of p38‐MAPK.^[^ ^41^ ^]^ Moreover, CBD exerts analgesic effect by binding to 5‐hydroxytryptamine 1A (5‐HT_1A_) receptors, then activating the 5‐hydroxytryptaminergic system in the spinal cord.^[^ ^42^ ^]^ In summary, CBD not only has an analgesic property but also slows the progression of diabetic neuropathy. Therefore, CBD has possible therapeutic advantages for diabetic neuropathy, which is worthy of further exploration and clinical verification.

### 2.5 Diabetic brain injury

Diabetes can lead to brain inflammation, cortical and subcortical atrophy, white matter abnormalities, cerebrovascular changes, blood‐brain barrier disruption, altered synaptic plasticity in the hippocampus, calcium imbalance, and overall dysregulation of brain metabolism,^[^ ^43^, ^44^, ^45^ ^]^ which cause diabetes‐related brain damage, such as hyperglycemia‐induced cognitive impairment, diabetic Alzheimer's disease, and diabetic stroke. No relevant investigations have yet demonstrated the potential positive effects of CBD on animal models of diabetes or human cognitive decline. It is interesting to note that CBD has shown potential in mitigating cognitive deficits in preclinical research of inflammation‐induced cognitive dysfunction,^[^ ^46^, ^47^, ^48^ ^]^ cerebral ischemia,^[^ ^49^, ^50^, ^51^, ^52^, ^53^ ^]^ and hepatic encephalopathy.^[^ ^54^, ^55^, ^56^ ^]^ These studies suggest that CBD may have neuroprotective effects and could potentially be used as a therapeutic agent for these conditions. While preclinical studies have shown promising effects regarding the potential therapeutic effects of CBD for cognitive deficits, particularly in neurodegenerative diseases, there is still a need for further research to determine whether CBD can be effective in treating cognitive impairment caused by diabetes. Human clinical trials are necessary to confirm these findings and establish the safety and efficacy of CBD as a treatment option for diabetes‐induced cognitive impairment.

Owing to its antioxidant, anti‐inflammatory and neuroprotective properties, CBD has been proposed as a promising drug for the treatment of Alzheimer's disease.^[^ ^57^ ^]^ CBD has been found to inhibit acetylcholinesterase activity,^[^ ^58^ ^]^ and significantly increase central acetylcholine content, and maintain the function of residual cholinergic neurons. Moreover, CBD is able to inhibit the deposition and expression of amyloid‐β peptides and exert anti‐apoptotic activity through selective activation of peroxisome proliferator‐activated receptor γ (PPAR‐γ).^[^ ^59^ ^]^ In parallel, CBD can increase the clearance of amyloid‐β peptides^[^ ^60^ ^]^ and exhibit anti‐inflammatory and anti‐gliosis properties.^[^ ^61^, ^62^ ^]^ Furthermore, CBD may activate PI3K/AKT signaling via transient receptor potential vanilloid type 1 (TRPV1) receptors, which inactivates glycogen synthase kinase 3‐β (GSK3β) via serine phosphorylation and attenuates tau protein phosphorylation.^[^ ^63^, ^64^ ^]^

Diabetes significantly increases the incidence of stroke with the risk being 2–4 times higher than that in the non‐diabetic population,^[^ ^65^ ^]^ and the additional risk of stroke increases by 3% for each year of diabetic duration.^[^ ^66^ ^]^ A growing number of preclinical studies have shown that CBD has a significant therapeutic value in experimental ischemic stroke.^[^ ^53^, ^67^, ^68^, ^69^, ^70^ ^]^ In a mouse model of middle cerebral artery obstruction, CBD can reduce the extent of cerebral infarction, increase cerebral blood flow, alleviate neurological deficits, and decrease the permeability of the blood‐brain barrier.^[^ ^67^, ^68^, ^69^ ^]^ Moreover, in a rat model of perinatal arterial ischemic stroke, CBD can reduce brain injury and improve long‐term functional recovery.^[^ ^70^ ^]^ Furthermore, in a mouse model of cerebral ischemia caused by bilateral common carotid artery occlusion, CBD administration contributed to overall functional recovery after ischemic injury, while also reducing hippocampal neurodegeneration, white matter damage, and glial response.^[^ ^53^ ^]^ Taken together, the above findings suggest that CBD has a therapeutic effect on diabetes‐induced ischemic stroke, which is warranted for further validation in experimental animal models and clinical trials.

## 3 STRATEGIES FOR THE DISCOVERY OF THE TARGET(S) OF CBD

CBD has demonstrated potential therapeutic benefits for diabetes and its complications, but the specific molecular targets responsible for these effects are not yet fully understood. Identifying these targets is crucial for understanding how CBD works and for developing more effective treatments. As a result, discovering drug targets for CBD has become a key challenge in current CBD research. Activity‐based protein profiling (ABPP) and thermal proteome profiling (TPP) are valuable tools for identifying potential drug targets in drug development.^[^ ^71^, ^72^ ^]^ While they share some similarities, they have distinct advantages and limitations. ABPP relies on the availability of a suitable probe that can selectively label the target protein of interest. In contrast, TPP does not require compound labeling. However, the proteome analysis of TPP is much more complex than that of ABPP. Both ABPP and TPP can be used in complementary ways to provide a more comprehensive understanding of potential drug targets.^[^ ^71^, ^72^ ^]^

### 3.1 Activity‐based protein profiling (ABPP)

**FIGURE 2:**
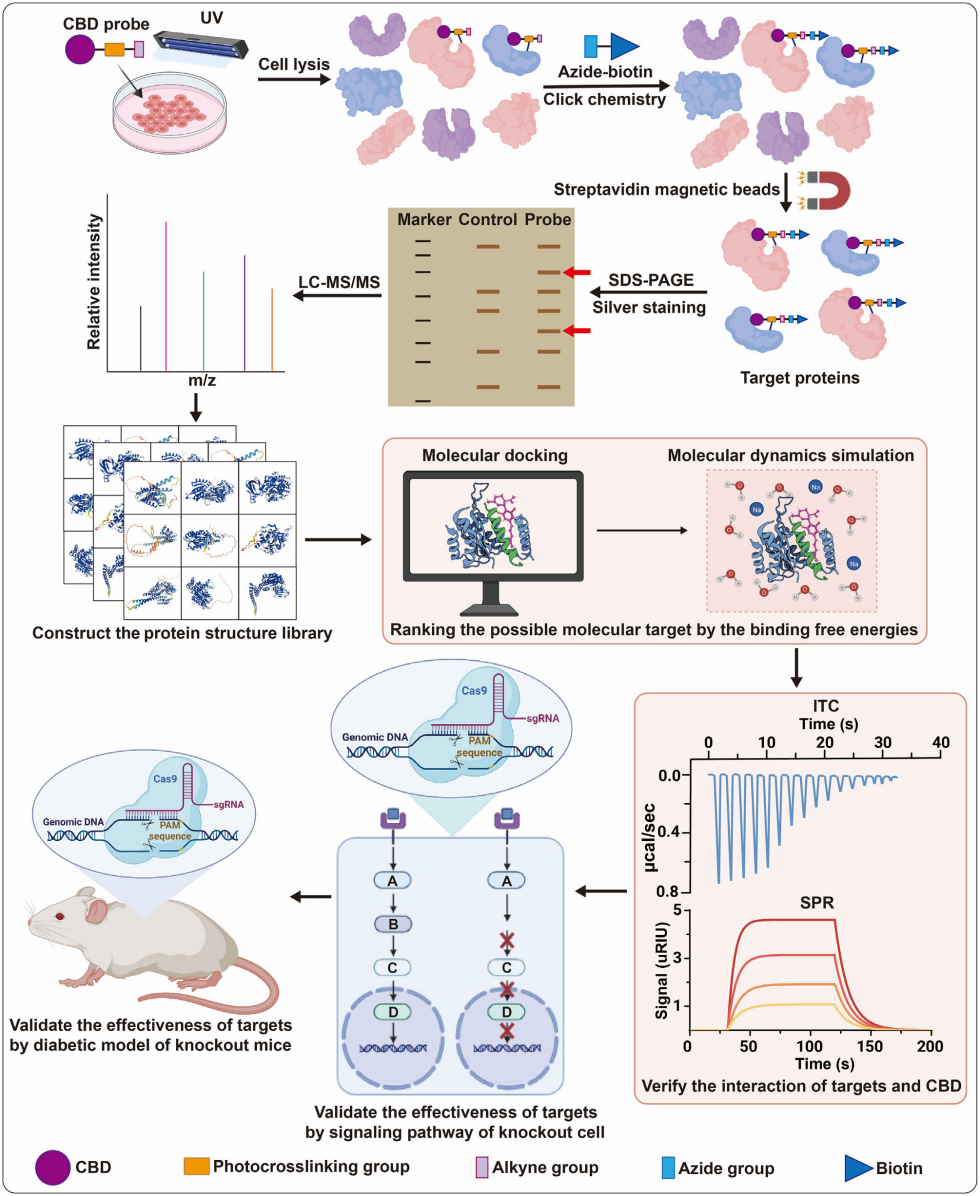
Strategy for the discovery of the targets of CBD by activity‐based protein profiling.

ABPP is an effective strategy for systematically dissecting the protein target(s) of small molecules (**Figure** **2**).^[^ ^73^, ^74^ ^]^ In order to discover the molecular target(s) of CBD, a CBD probe will be designed. The CBD probe is a specially designed molecule that contains three key components: (1) CBD pharmacophore, which is the part of the molecule responsible for binding to its target protein(s) in the cell. (2) Photo‐crosslinking group, which is a chemical moiety that can form a covalent bond with its neighboring molecules upon exposure to UV light. This allows the probe to irreversibly attach to the target protein, capturing it for further analysis. (3) Alkyne group, which is a chemical handle that enables the probe to be selectively labeled with other molecules using click chemistry.

To capture the proteins that directly interact with the CBD probe, the probe is incubated with cells derived from tissues in diabetic model animals. Upon UV irradiation, the photo‐crosslinking group of the probe covalently reacts with the target protein. The cells are then lysed to obtain whole‐cell protein extracts. To efficiently and specifically attach the biotin molecule to the CBD probe, the alkyne group on the probe is covalently linked to azide‐biotin using click chemistry. This biotin molecule acts as a “handle” for magnetic bead separation, which allows for the isolation of the target proteins from the protein extracts. Finally, the isolated target proteins are identified using liquid chromatograph mass spectrometer/mass spectrometer (LC‐MS/MS), which provides information about the identity and quantity of the proteins present.

To investigate the potential targets of CBD, protein structures can be obtained by either retrieving them from the Protein Data Bank or predicting them using computational methods such as RoseTTAFold or Alphafold2. Molecular docking and molecular dynamics simulations can then be performed to assess the binding of CBD to each protein. The resulting data can be used to rank the possible molecular targets of CBD based on their binding free energies. Prioritizing proteins in this way can help to identify the most promising candidates for further study. To verify the interactions between the target protein and CBD, biophysical binding characterizations such as isothermal titration calorimetry and surface plasmon resonance can be used. Additionally, the effectiveness of the target can be evaluated by using gene knockout strategies on cells and model animals. The discovery of novel CBD drug targets will guide the future development of CBD‐based therapeutics.

### 3.2 Thermal proteome profiling (TPP)

**FIGURE 3:**
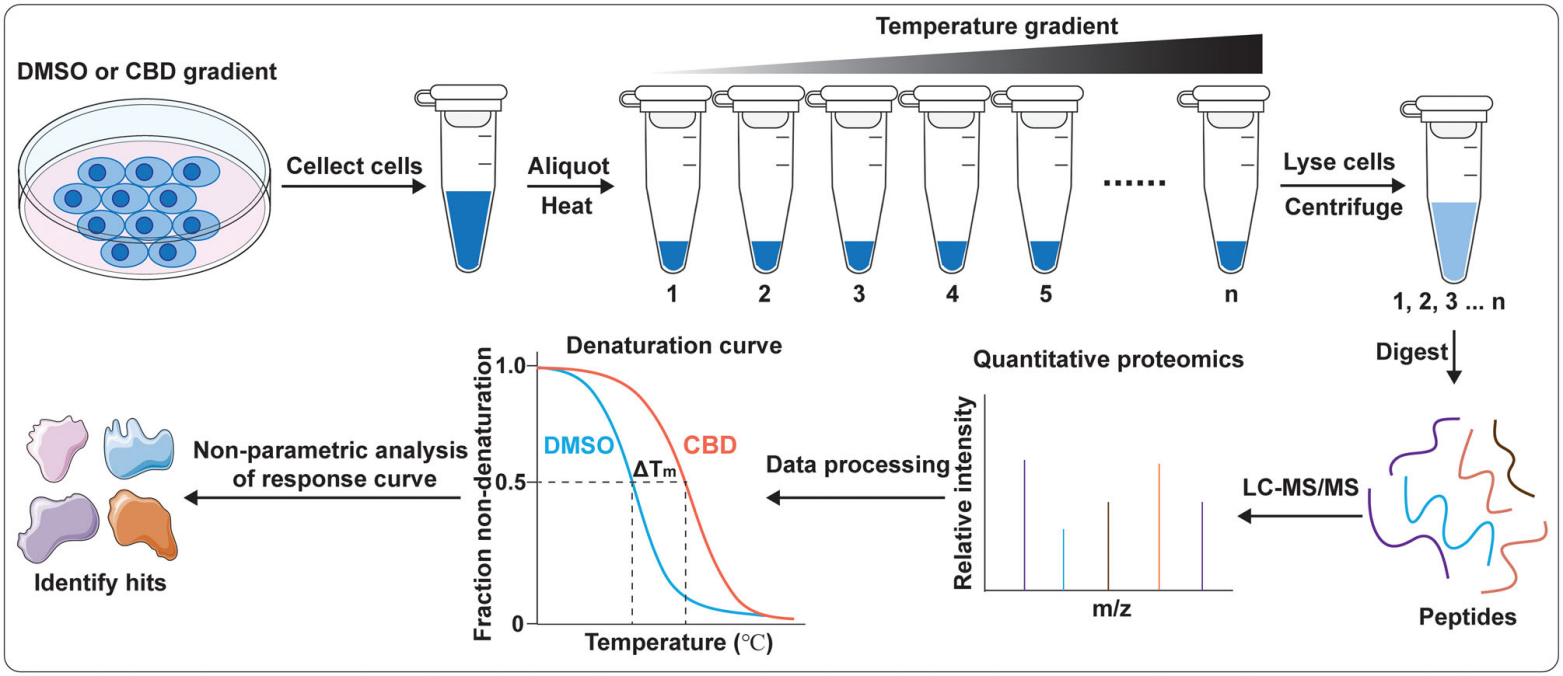
Strategy for the discovery of the targets of CBD by thermal proteome profiling.

In addition to ABPP, thermal proteome profiling (TPP) (**Figure** **3**) is a proteomic alternative that directly probes the physical interaction between the ligand and the target protein, and has recently emerged as a powerful tool for target discovery in drug development.^[^ ^75^, ^76^ ^]^ By comparing the abundance of individual proteins across a range of temperatures, it is possible to construct a thermal stability profile for each protein. Upon ligand binding, proteins undergo thermal stabilization or destabilization. To perform TPP, cells are exposed to different concentrations of the ligand and subjected to a range of temperatures, leading to the thermal denaturation of proteins. Soluble protein is then extracted and quantified using multiplexed, quantitative mass spectrometry, producing thousands of thermal denaturation profiles. By analyzing the ligand‐dependent thermal shift, it is possible to identify proteins that interact with the ligand. Once a potential target has been identified, further experiments should be conducted as following the ABPP strategy in 3.1 to validate its role in the process of interest.

## 4 RATIONAL DRUG DESIGN TO EXPLORE THE STRUCTURE–ACTIVITY RELATIONSHIP (SAR) OF CBD

### 4.1 Progress in the clinical development of CBD

Clinical development of CBD has been progressing rapidly in recent years. Epidiolex, a CBD‐based drug developed by GW Pharmaceuticals, was approved by the Food and Drug Administration (FDA) in 2018 for the treatment of seizures associated with two rare forms of epilepsy: Lennox‐Gastaut syndrome and Dravet syndrome.^[^ ^77^ ^]^ This approval marked a significant milestone for CBD as it became the first FDA‐approved drug derived from cannabis. Apart from Epidiolex, several other CBD‐based drugs are currently in clinical trials. For instance, Zynerba Pharmaceuticals is developing a CBD‐based gel for the treatment of Fragile X syndrome;^[^ ^78^, ^79^ ^]^ Additionally, GW Pharmaceuticals has developed a CBD‐based drug Sativex, which is used to treat multiple sclerosis‐related spasticity in several countries outside the US.^[^ ^80^ ^]^ Although CBD is safe and generally well tolerated as plant‐derived polyphenol, the therapeutic applications of CBD are greatly limited by its strong lipophilicity and poor oral bioavailability. To overcome this major limitation, a water‐soluble CBD formulation might offer a better therapy for a wide range of medical conditions. Incorporating CBD into a novel drug delivery system is hopefully to boost its bioavailability, prolong its half‐life, and improve its efficacy. With the flourishing development of delivery technologies by targeting specific organelles,^[^ ^81^, ^82^, ^83^ ^]^ such as mitochondria, it may be possible to further enhance the therapeutic index of CBD while minimizing its toxicity.

### 4.2 Rational drug design to explore the SAR of CBD

**FIGURE 4:**
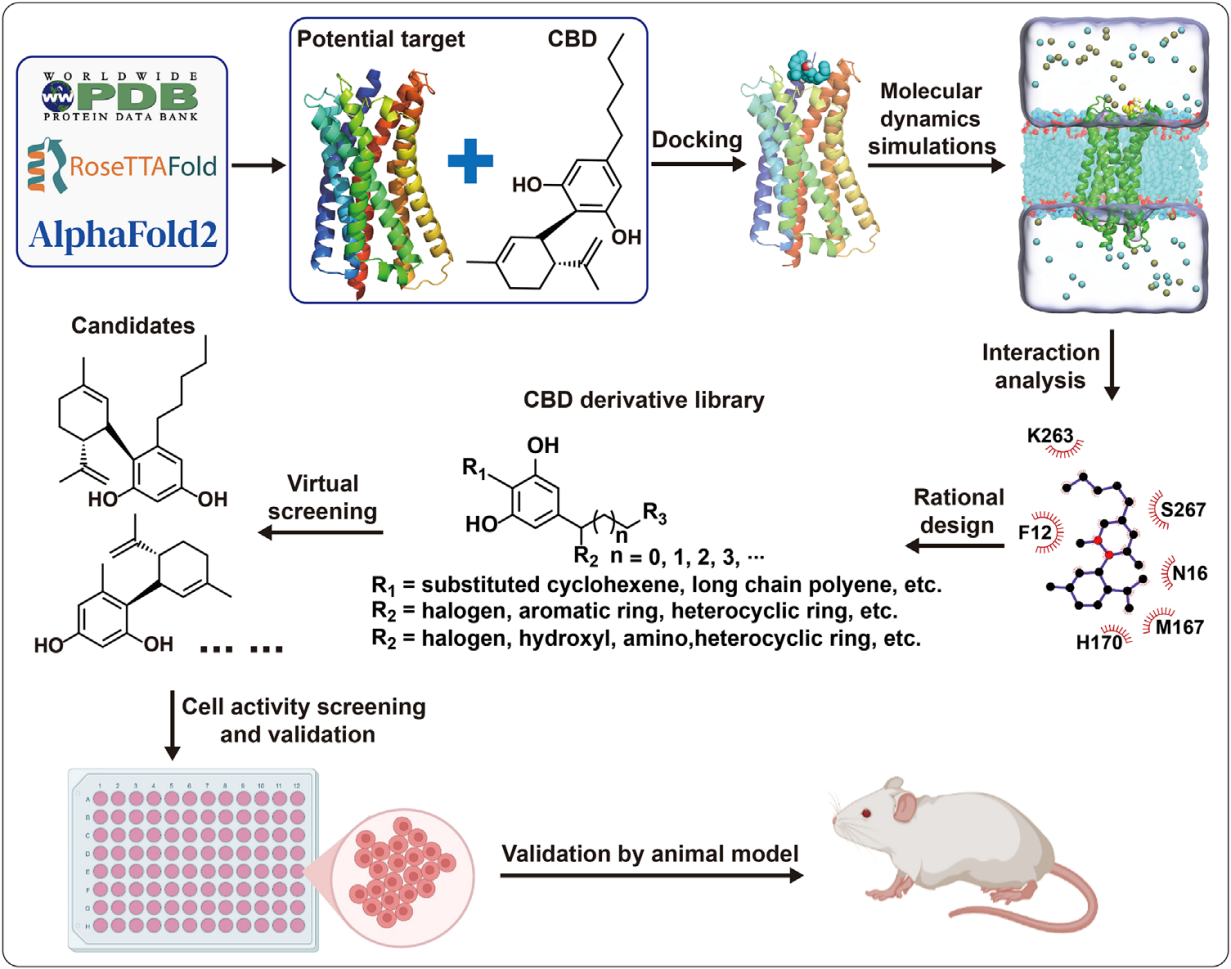
Schematic diagram of CBD structure optimization process.

In order to develop a more effective therapy, structure‐activity relationships (SARs) would be another feasible strategy, which allows one to rationally explore chemical space and develop a chemical series with significantly improved potency, reduced toxicity, and sufficient bioavailability. Considering that the subtle structural differences among CBD and CBD analogues could lead to contrasted activities,^[^ ^84^, ^85^ ^]^ the SAR of CBD for the treatment of diabetes and diabetic complications is worthy of exploration. Therefore, structure‐based rational drug design would be an attractive strategy for the exploration of CBD SAR (**Figure** **4**). The structures of potential target proteins are obtained from the Protein Data Bank or predicted by RoseTTAFold or Alphafold2. The next step is to dock CBD onto the potential target and perform molecular dynamics simulations to relax the binding pose. By analyzing the interactions between the pharmacophores of CBD and key residues during simulations, we can rationally design a library of CBD derivatives and use it for virtual screening to identify promising candidates. These candidates can then be validated in vitro and in vivo using cell activity assays and animal models, respectively. It should be noted that CBD has poor oral bioavailability owing to its strong lipophilicity, which seriously limits its therapeutic applications. Therefore, the pharmacokinetics of the potential hits should be evaluated. Alternatively, a water‐soluble formulation or delivery strategy might offer a better treatment effect. It should be noted that the use of CBD has been limited by legal and regulatory hurdles. However, the lead compound derived from CBD could overcome these barriers.

## 5 CONCLUSIONS

Diabetes is the seventh most common disease affecting human health and can affect multiple organs in the body, leading to target organ damage. The treatment of diabetic uropathy and its complications is a major challenge. The current clinic treatment is still focused on glycemic control and symptomatic drug therapy for target organ damage. However, no drugs are available for the disease itself or its complications. CBD is a non‐psychoactive cannabinoid, which has demonstrated great translational potential. According to the current experimental results, CBD is of great value in the treatment of diabetes and its complications. CBD can improve pancreatic islet function, reduce pancreatic inflammation and improve insulin resistance. For diabetic complications, CBD not only has a preventive effect but also has a therapeutic value for existing diabetic complications and improves the function of target organs. However, the safety and effectiveness of CBD are still needed to prove. It should be acknowledged that the clinical application of CBD in the treatment of diabetes and its complications has a long way to go. The dissecting of the pharmacology and therapeutic role of CBD in diabetes would guide the future development of CBD‐based therapeutics for treating diabetes and diabetic complications.

## AUTHOR CONTRIBUTIONS

Jin Zhang, Cong Lin, Sha Jin, Hongshuang Wang, Yibo Wang, and Huiying Zhao wrote the manuscript. Xiubo Du, Mark R. Hutchinson, Huiying Zhao, and Le Fang edited and reviewed the paper. Xiaohui Wang designed the research, edited the manuscript, and gave the final approval of the paper.

## CONFLICT OF INTEREST STATEMENT

The authors declare that no conflicts of interest.
